# Senescence chips for ultrahigh‐throughput isolation and removal of senescent cells

**DOI:** 10.1111/acel.12722

**Published:** 2018-01-16

**Authors:** Yuchao Chen, Pan Mao, Antoine M. Snijders, Daojing Wang

**Affiliations:** ^1^ Newomics Inc. Emeryville CA USA; ^2^ Lawrence Berkeley National Laboratory Berkeley CA USA

**Keywords:** anti‐aging, cellular senescence, mesenchymal stem cells, microfluidic chip, size separation, total body irradiation

## Abstract

Cellular senescence plays an important role in organismal aging and age‐related diseases. However, it is challenging to isolate low numbers of senescent cells from small volumes of biofluids for downstream analysis. Furthermore, there is no technology that could selectively remove senescent cells in a high‐throughput manner. In this work, we developed a novel microfluidic chip platform, termed senescence chip, for ultrahigh‐throughput isolation and removal of senescent cells. The core component of our senescence chip is a slanted and tunable 3D micropillar array with a variety of shutters in the vertical direction for rapid cell sieving, taking advantage of the characteristic cell size increase during cellular senescence. The 3D configuration achieves high throughput, high recovery rate, and device robustness with minimum clogging. We demonstrated proof‐of‐principle applications in isolation and enumeration of senescent mesenchymal stem cells (MSCs) from undiluted human whole blood, and senescent cells from mouse bone marrow after total body irradiation, with the single‐cell resolution. After scale‐up to a multilayer and multichannel structure, our senescence chip achieved ultrahigh‐throughput removal of senescent cells from human whole blood with an efficiency of over 70% at a flow rate of 300 ml/hr. Sensitivity and specificity of our senescence chips could be augmented with implementation of multiscale size separation, and identification of background white blood cells using their cell surface markers such as CD45. With the advantages of high throughput, robustness, and simplicity, our senescence chips may find wide applications and contribute to diagnosis and therapeutic targeting of cellular senescence.

## 1 INTRODUCTION

Cellular senescence is a state of permanent cell cycle arrest due to genotoxic stresses and has been shown to be involved in organismal aging and tumorigenesis (Campisi & d'Adda di Fagagna, 2007; Munoz‐Espin & Serrano, 2014). Therefore, senescent cell is an important biomarker for aging as well as genotoxic stresses such as ionizing radiation. However, the small number of senescent cells in biofluids such as whole blood limits their quick and sensitive detection. An effective isolation approach is highly desired for senescent‐cell‐based point‐of‐care diagnostics such as radiation biodosimetry. Moreover, recent animal studies have demonstrated the potential of therapeutic targeting of senescent cells for anti‐aging and age‐related diseases (Baar et al., 2017; Baker et al., 2016; Chang et al., 2016). Because pathways up‐ or downregulated in senescent cells, such as those involving p16, p21, and p53, also function at various degrees in their healthy counterparts throughout the tissues and organs (Kruiswijk, Labuschagne & Vousden, 2015; Munoz‐Espin & Serrano, 2014), conventional methods by targeting these pathways with small molecules and protein drugs could result in side effects in humans. Alternatively, physical means by taking advantage of the cell size increase during cellular senescence provides an attractive novel approach to selectively remove senescent cells from their nonsenescent counterparts and other background cells.

Different microfluidic techniques have been developed for cell separation based on their physical properties (size, deformability, density, etc.), including filtration, deterministic lateral displacement (DLD), inertial flow, and acoustofluidics (Chen et al., 2014; Shields, Reyes & Lopez, 2015; Wu, Chen & Lin, 2017; Xavier, Oreffo & Morgan, 2016). Among those techniques, filtration is the most promising approach to process undiluted whole blood for rare cell separation, and easily scaled up for high throughput (Lin et al., 2010). However, several challenges need to be overcome before this technique could be widely used. In dead‐end flow filtration which has the flow direction perpendicular to the filter surface, a common issue is the clogging and saturation of the filter, resulting in low separation efficiency, sample purity, and device robustness (Linkhorst, Beckmann, Go, Kuehne & Wessling, 2016). In some studies, a periodic reversed flow or fluidic oscillation was adopted to address clogging (McFaul, Lin & Ma, 2012; Yoon et al., 2016). These additional steps are helpful but drastically reduce the separation throughput and operation simplicity. Another issue for filtration is that cell integrity is decreased as they squeeze through the filtration pores, which could result in the changes in cell cytoskeleton (Zheng et al., 2011). To avoid cell damage and clogging issue, cross‐flow filtration in microfluidics was developed with a flow direction parallel to the filter surface (Chen, Cui, Liu & Li, 2008; Sethu, Sin & Toner, 2006). Therefore, a shear force was generated to bring the bigger particles to the downstream instead of entering the filtration pores. However, to ensure effective cell separation in a parallel‐flow configuration, the cross‐flow filtration typically has a much longer channel with a throughput usually lower than 1 ml/hr. Despite the inherent low throughput for microfluidic devices, a higher throughput (e.g., >1 ml/min) is highly desired to process a large volume of whole blood samples (Loutherback et al., 2012; Zheng et al., 2011). High throughput is particularly challenging for a continuous flow because of the difficulties in system integration and fluidic control for multiplexing on a microfluidic chip.

To overcome the clogging and cell damage issue while still achieve a high throughput and recovery rate, we developed a microdevice (senescence chip) for three‐dimensional size sieving by taking advantages of both dead‐end flow and cross‐flow filtrations. A slanted micropillar array was fabricated with an inclination angle relative to the fluidic flow (between 0° to 90°). Therefore, the particles could not only be sieved efficiently but also experience a fluidic shear force to reduce clogging and preserve cell integrity. Moreover, the micropillars worked as cantilevers, which had only one end fixed. Their flexibility allowed small deformation when experiencing a fluidic pressure, creating hundreds of shutters in the vertical direction responsive to the flow rate. These shutters helped to release backpressure, reduce clogging, and dramatically improve separation throughput.

We utilized our senescence chip to isolate and analyze senescent cells in undiluted whole blood and mouse bone marrow. We chose mesenchymal stem cells (MSCs) because we have previously characterized their ionizing radiation‐induced senescence progression (Wang & Jang, 2009). In this study, we utilized H_2_O_2_‐ and *X*‐ray‐induced senescent human MSCs spiked in whole blood, as a model biological system, to demonstrate the rapid separation and analysis of senescent cells using our senescence chip. The optimized device was then used for an animal study to isolate senescent cells from the bone marrow of mice undergone total body irradiation (TBI) of *X*‐ray. To achieve ultrahigh‐throughput removal of senescent cells for blood purification, we enlarged the chip dimensions and stacked multiple chips to build a multiplexed system. We demonstrated that our scaled‐up senescent chip could achieve a parallel processing with a throughput up to 300 ml/hr.

## 2 RESULTS AND DISCUSSION

### 2.1 Design of senescence chips

**Figure 1:**
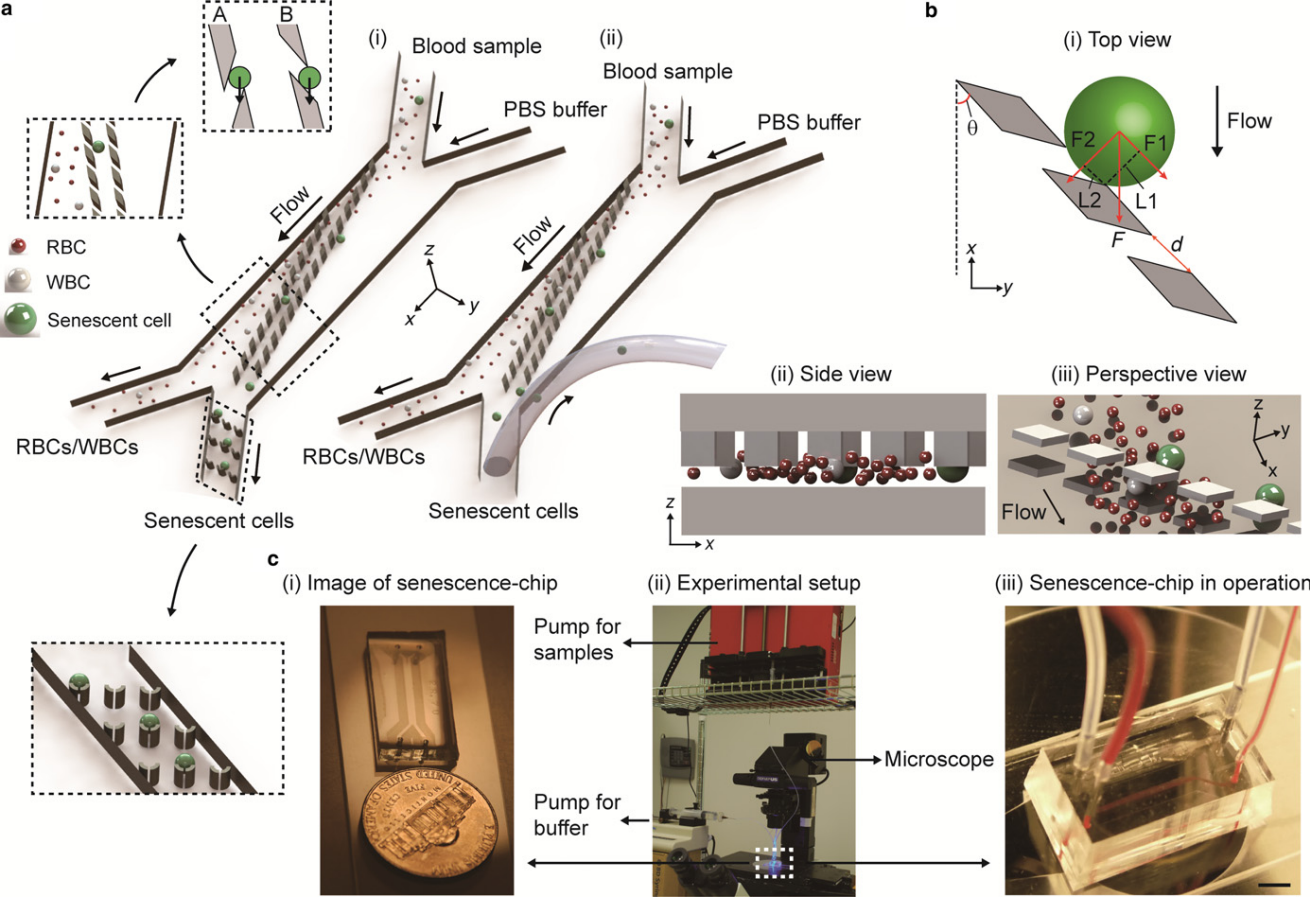
Design and working mechanism of senescence chip. (a) Illustration of two types of chips: (i) a chip with a 3D filter array and a cell trap array for capture and single‐cell analysis of senescent cells in blood; and (ii) a chip with a 3D filter array connected with a tubing at the outlet for removal of senescent cells from blood. Zoom‐in regions show schematic separation and trapping of RBC, WBC, and senescent cells, along with two types of pillar shapes A and B. (b) Mechanism of size‐based cell separation with a 3D filter array: (i) the top view of the filters with force analysis on the *x* and *y* directions; (ii) the side view of the filters on the *x* and *z* directions; and (iii) the perspective view of the filters on the *x*,* y*, and *z* directions. (c) Images of the experimental setup and operation: (i) an actual‐size image of a senescence chip relative to a US dime; (ii) the experimental setup showing tubing connections and pumps; and (iii) a senescence chip in operation of processing whole blood samples. Scale bar represents 5 mm in (c‐iii). RBC: red blood cell; WBC: white blood cell

We first developed the senescence chips, which monolithically integrate two rows of tilted 3D filter array for size‐based cell separation with all necessary inlets and outlets for samples and buffers (Figure 1). Two types of senescence chips were designed for different purposes. For analysis of senescent cells in small volumes of whole blood or bone marrow, the senescence chip contains a 3D‐filter array to isolate MSCs, followed with a cell trap array to capture MSCs after separation for enumeration and single‐cell analysis of senescent cells (Figure 1a‐i). For rapid removal of senescent cells from whole blood, the senescence chip does not contain cell traps, but the chip outlet is connected directly to a tubing to remove senescent cells from whole blood (Figure 1a‐ii). The other end of the tubing goes to a waste or a collection tube for further analysis if needed.

We performed modeling to optimize the design of our chips (Figure 1b, and Appendix S1 for equations and parameters). A 3D filter array contained micropillars inside a channel to achieve cell separation on the *x‐y* plane as well as in the *z*‐direction. During the separation of MSCs from whole blood, RBCs and WBCs can easily pass through the filter from both the *z*‐direction and *x‐y* plane, while the MSCs with a larger size will not cross the filter but instead roll down. The optimized design of our 3D filter array could reduce the system backpressure, reduce clogging of the filter, and improve the throughput.

Figure 1c shows the experimental setup for the operation of our senescence chip. Two syringe pumps are used to deliver the 1 × PBS buffer and blood samples into two inlets, respectively (Figure 1c‐ii). A sheath flow of 1 × PBS buffer ensures the blood sample flow into the left outlet. When the cells in the blood sample flow down to the main channel, small cells such as RBCs and WBCs pass the 3D filter array without changing their flow path, as a result, exiting to the left outlet. However, larger cells such as MSCs are filtered out by the filters and roll down following the pillars to the right outlet (Figure 1c‐iii).

### 2.2 Operation of senescence chips

**Figure 2:**
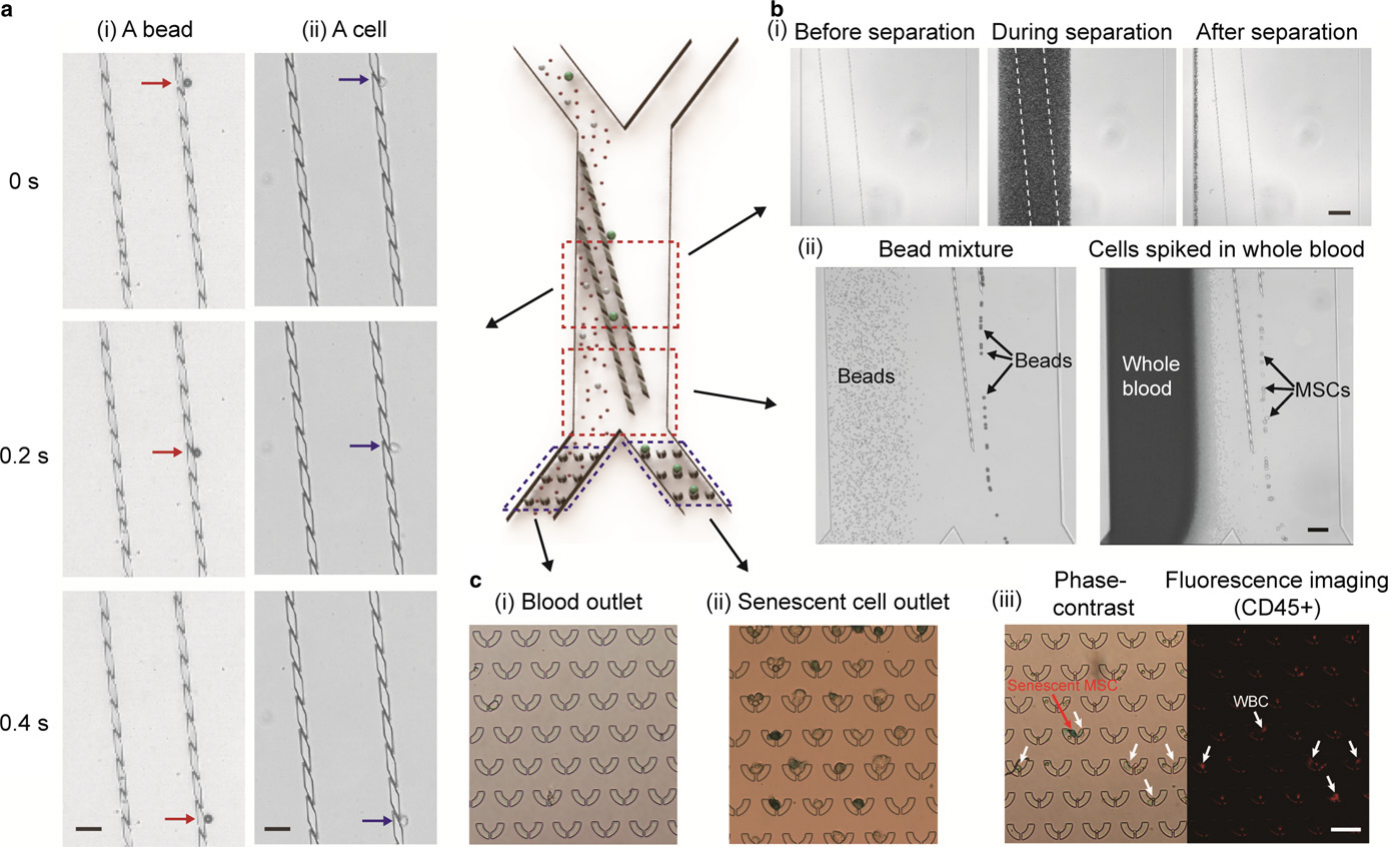
Operation of senescence chip. (a) Time‐lapse images showing: (i) a bead and (ii) a cell, roll down on a 3D filter array. (b) (i) Images showing undiluted whole blood passes through a 3D filter array without clogging; (ii) Stacking images showing complete separation of 18‐μm beads from 10‐μm beads (left), and separation of mesenchymal stem cells (MSCs) from undiluted whole blood (right). (c) Images of cell trap array located at: (i) blood outlet and (ii) senescent‐cell outlet, after separation of MSCs from whole blood. Cells with blue color are senescent cells (SA‐β‐gal positive); (iii) Phase‐contrast and fluorescence imaging of CD45 labeling for identification of senescent MSCs and WBCs. Scale bars represent 50 μm in (a) and (c), 150 μm in (b**‐**i), and 100 μm in (b‐ii), respectively. (a)‐(c) are shown as the corresponding zoom‐in regions on a schematic senescence chip for presentation clarity

We next tested the performance of our senescence chip (Figure 2). On a 4‐μm 3D filter array (*d* = 4 μm), time‐lapse images clearly demonstrated that a 15‐μm bead and a MSC rolled down on the filter array (Figure 2a). In addition, when undiluted whole blood sample passed through the 3D filter array, no clogging was observed (Figure 2b‐i). We further verified the separation ability of the senescence chip with mixtures of polystyrene microbeads and MSCs spiked into undiluted fresh human whole blood (Figure 2b‐ii). When 10‐μm and 18‐μm beads flowed down the channel, only the 18‐μm particles were filtered out by the filter while the smaller beads crossed the filter, as shown in the stacking image of Figure 2b‐ii (left) and Video S1. After filtration, the 18‐μm particles moving along the filter array were collected downstream from the right outlet. Similarly, the 4‐μm 3D filter could isolate MSCs from blood cells, as shown in the stacking image of Figure 2b‐ii (right) and Video S2. Although basal and senescent human MSCs are heterogeneous in size, our filter can still isolate most of them from blood cells due to their larger average sizes than RBCs and WBCs. After separation, MSCs were captured by the cell trap array located at the device outlet (Figure 2c). On‐chip SA‐β‐gal staining showed good separation of senescent MSCs (blue, right outlet) and almost no background MSCs in blood cells (left outlet) (Figure 2c‐i and ii). After labeled with anti‐CD45 antibodies, background WBCs were able to be identified and excluded by comparing the phase‐contrast image to fluorescence image (Figure 2c‐iii).

### 2.3 Validation of senescence chips

**Figure 3:**
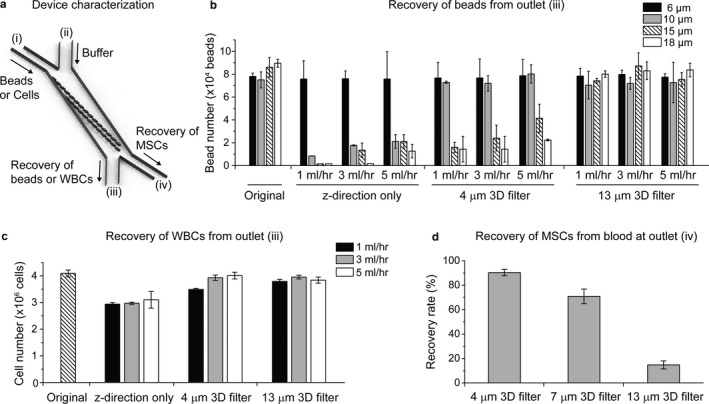
Validation of senescence chip for size‐based separation. (a) Schematic of a senescence chip for characterization with beads or cells. (b) Recovery of beads from outlet (iii), for four sizes of beads (6 μm, 10 μm, 15 μm, and 18 μm) mixed to characterize three types of senescence chips (*z*‐direction only filter, 4‐μm 3D filter, and 13‐μm 3D filter) at three flow rates (1 ml/hr, 3 ml/hr, and 5 ml/hr). (c) Recovery of WBCs isolated from whole blood from outlet (iii), with three types of senescence chips at three flow rates as in (b). (d) Recovery of basal mesenchymal stem cells (MSCs) from undiluted whole blood at outlet (iv), with three types of senescence chips at a flow rate of 3 ml/hr. WBCs: white blood cells

To further validate the separation ability of our senescence chip and its dependence on 3D filter sizes and flow rates, we performed device characterization using bead mixtures, isolated WBCs, and basal MSCs spiked in whole blood (Figure 3). We input the mixture of beads and cells from the inlet (i) and recovered the beads and cells, which passed through the filter, from both the outlet (iii) and (iv) (Figure 3a), and measured their numbers. The bead mixture contained roughly an equal mix of four different sizes (6 μm, 10 μm, 15 μm, and 18 μm), and the total numbers of beads for each size were around 8.0 – 9.0 × 10^4^. As shown in Figure 3b, most of the beads larger than 10 μm were removed by the z‐direction only filter array (dam‐like, with no openings in the x and y directions), while only 6‐μm beads could pass the filter. As the flow rate increased from 1 ml/hr to 5 ml/hr, the number of beads larger than 10 μm also slightly increased, but was still lower than 25% of the original concentration. The results indicate that in our 3D filter array, the spacing along the z‐direction was smaller than 10 μm, but it could increase as the flow rate increased. With the 4‐μm 3D filter array, more than 90% of the 6‐μm and 10‐μm beads could pass through the filter to outlet (iii), while larger particles were removed, suggesting the effective spacing along the *z*‐direction was increased in the presence of openings in the *x* and *y* directions. As the pillar spacing increased to 13 μm, all size of beads from 6 μm to 18 μm could be recovered from outlet (iii), independent of the flow rates. Majority of the WBCs have a size between 8 and 12 μm. To demonstrate our ability to recover WBCs while removing senescent MSCs from whole blood, we used the RBC‐lysed blood sample to test our devices. The original (input) cell number of WBCs was around 4 × 10^6^. As shown in Figure 3c, the z‐direction only filter array allowed ~75% of the WBCs to pass, while for the 4‐μm and 13‐μm 3D filter arrays, almost all of the WBCs could pass through and be recovered from outlet (iii). No WBCs were observed on our cell traps at outlet (iv), confirmed by negative immunostaining with CD45. Presumably, the smaller WBCs were filtered through to the outlet (iii) or passed through the gap between the cell traps (~10 μm) at outlet (iv), while the giant WBCs were prefiltered by our 40‐μm cell strainer prior to on‐chip separation.

We also spiked basal MSCs in whole blood and the original input number of MSCs was approximately 1 × 10^4^. The number of recovered MSCs was measured at outlet (iv) and the recovery rate was calculated, which is defined as the ratio between the recovered MSC number and the input MSC number. As shown in Figure 3d, three types of 3D filter arrays with different filter sizes (pillar spacing of 4 μm, 7 μm, and 13 μm) were tested at a flow rate of 3 ml/hr. The recovery rate of basal MSCs at outlet (iv) dropped from ~90% to ~20% as the 3D filter size increased from 4 μm to 13 μm.

Based on the results we obtained, we chose 4‐μm 3D filter arrays for analysis of senescent cells, because this filter size could isolate most of the MSCs from whole blood containing most of RBCs and WBCs, which allowed us to quantify the numbers and percentage of senescent MSCs among the total MSCs. On the other hand, for removal of senescent cells from whole blood, we wanted to maximize the recovery of basal MSCs from outlet (iii) while only selecting senescent MSCs to outlet (iv); therefore, we used 13 μm filter size for this application.

### 2.4 Senescence chips for analysis of senescent cells in biofluids

**Figure 4:**
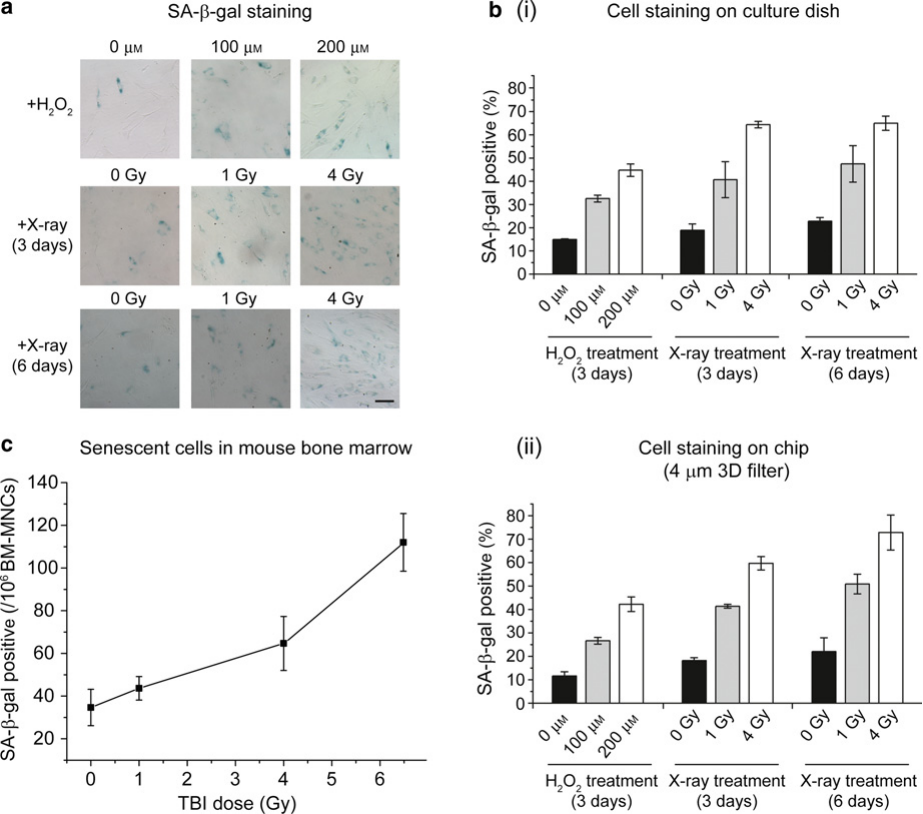
Application of senescence chip for analysis of senescent cells in biofluids. (a) SA‐β‐gal staining of mesenchymal stem cells (MSCs) cultured on a 12‐well plate. The MSCs were treated with different doses of hydrogen peroxide (H_2_O_2_, 0, 100, 200 μm) and *X*‐ray (0, 1, 4 Gy), and analyzed 3 days and 6 days after the treatments. Cells stained blue are SA‐β‐gal positive. SA‐β‐gal: senescence‐associated beta‐galactosidase. (b) Quantitation of SA‐β‐gal staining of MSCs on culture dish (i) and MSCs isolated from human whole blood on the senescence chip (ii). The percentage of SA‐β‐gal positive was calculated for the blue‐stained MSCs among the total MSCs. (c) Isolation and analysis of senescent cells from mouse bone marrow after TBI of 0, 1 Gy, 4 Gy, and 6.5 Gy X‐ray radiation (*n* = 4), respectively. TBI: Total body irradiation. Scale bar represents 100 μm in (a)

After validation, we demonstrated the applications of our senescence chips for analysis of senescent cells in human whole blood and mouse bone marrow samples (Figure 4). Mesenchymal stem cells have been demonstrated to undergo in vitro cellular senescence by treatment of hydrogen peroxide and irradiation of *X‐*ray (Wang & Jang, 2009). Mesenchymal stem cells were treated with different doses of hydrogen peroxide (H_2_O_2_, 0, 100, 200 μm) and *X*‐ray (0, 1, 4 Gy), and analyzed 3 days and 6 days after the treatments. We observed both dose‐dependent and day‐dependent increases of the percentage of SA‐β‐gal‐positive (stained blue) MSCs on 12‐well cell plates (Figure 4a and b‐i). The H_2_O_2_‐ and X‐ray‐induced senescent MSCs (~500 cells) were spiked in undiluted whole blood (~2 ml) and underwent separation on our senescence chip with a 4‐μm 3D filter array at 3 ml/hr and captured and stained on the single‐cell traps at outlet. We observed dose‐ and day‐dependent increases of the percentage of SA‐β‐gal‐positive MSCs on the cell trap, matching those determined by direct cell staining on culture dish (Figure 4b‐ii). Importantly, the quantitation by our senescence chip was achieved by starting with small numbers of MSCs and in the presence of undiluted human whole blood.

We also applied our senescence chip to isolate senescent cells from mouse bone marrow. Four groups of mice were exposed to different doses of *X*‐ray (0, 1, 4, 6.5 Gy); 10 days after TBI, their bone marrow was obtained and diluted into 1.5 ml with 1 × PBS. We aliquoted ~1 × 10^6^ bone marrow mononuclear cells (BM‐MNCs) from each sample and diluted them into 2 ml for cell separation directly on our chips. As shown in Figure 4c, the number of senescent cells isolated by our senescence chips increased from average 34 to 112 as IR dose increased. The results demonstrate that our senescence chip is able to isolate and enumerate senescent cells from small volumes of various biofluids.

### 2.5 Senescence chips for removal of senescent cells from whole blood

**Figure 5:**
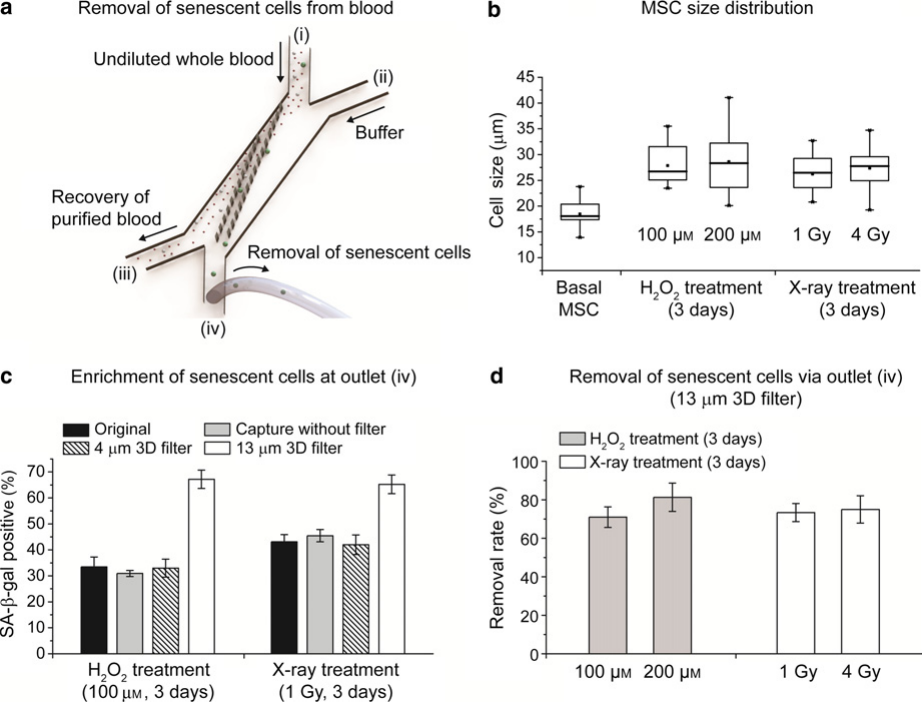
Application of senescence chip for removal of senescent cells from whole blood. (a) Schematic of removal of senescent cells from whole blood, using a 13‐μm 3D filter. (b) Cell size distribution of basal mesenchymal stem cells (MSCs) and senescent MSCs, 3 and 6 days after treatment with different doses of hydrogen peroxide and *X*‐ray. (c) Enrichment of senescent cells at outlet (iv) using a 13‐μm 3D filter senescence chip. In comparison, original MSCs without separation, MSCs directly captured on a cell trap array without a 3D filter, and MSCs processed on a senescence chip with a 4‐μm 3D filter were also studied. (d) Removal of senescent cells from undiluted whole blood using a 13‐μm 3D filter senescence chip via outlet (iv). SA‐β‐gal: senescence‐associated beta‐galactosidase

**Figure 6:**
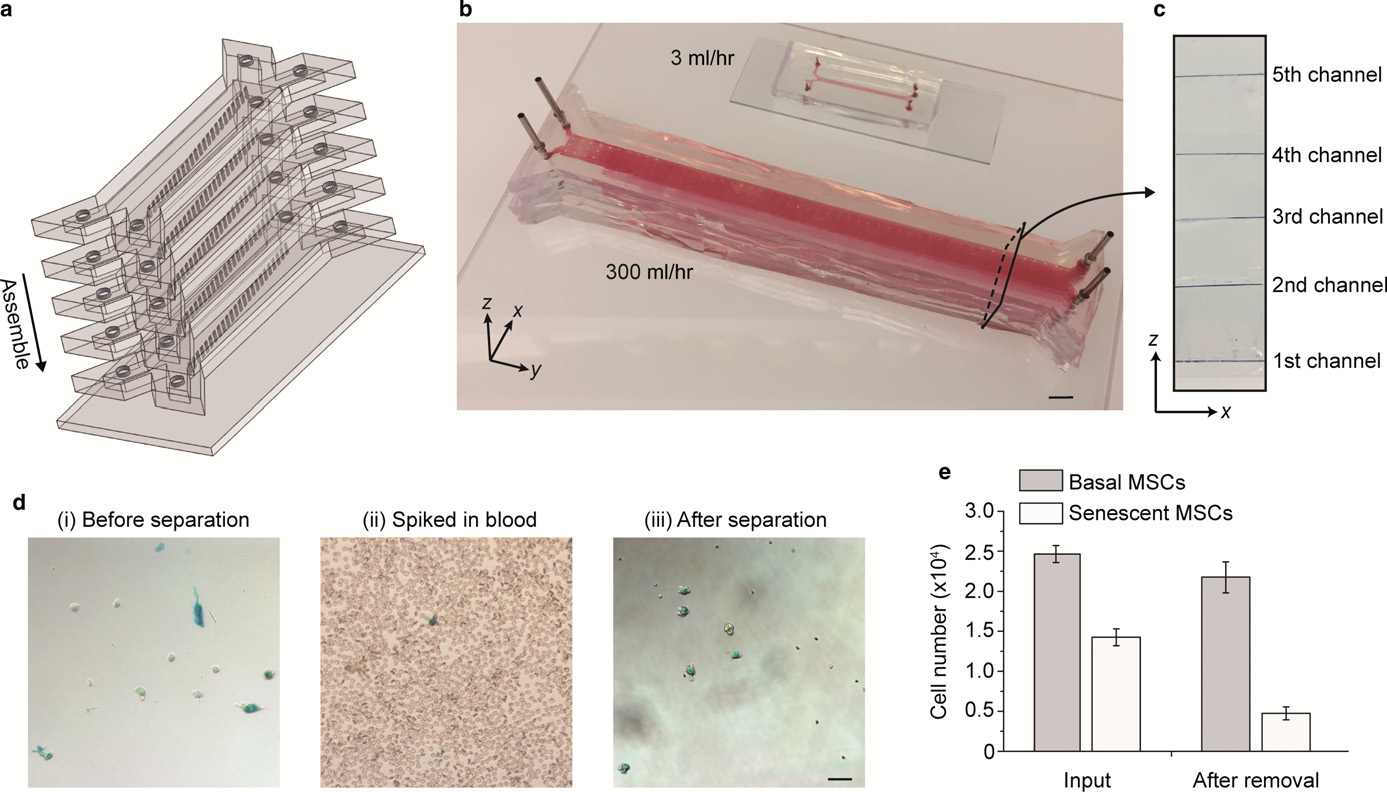
Ultrahigh‐throughput senescence chip for removal of senescent cells from human whole blood. (a) Schematic of the high‐throughput senescence chip. Five large‐dimension channels are stacked and integrated for parallel processing. (b) Image of a high‐throughput senescence chip compared to a regular‐size single‐unit device. (c) Cross‐sectional view of the multilayer and multichannel senescence chip showing integration of five channels in five vertical layers. (d) Microscope images showing mesenchymal stem cells (MSCs) before separation, spiked in blood, and after separation. (e) Quantification of the numbers of basal MSCs and senescent MSCs spiked into whole blood, before and after removal of senescent MSCs using our senescence chip. Scale bars represent 10 mm in (b) and 50 μm in (d)

We further demonstrated the applications of our senescence chips for removal of senescent cells from whole blood samples for potential therapeutic targeting of cellular senescence (Figure 5 and Figure 6). The overall strategy was to maximize recovery of the major blood components including plasma, RBCs, WBCs, and healthy cells (in this case basal MSCs) from the outlet (iii), while removing most of the senescent MSCs through the outlet (iv) (Figure 5a). To choose the optimal filter size of our 3D filter array for this application, we determined the average cell size of basal MSCs to be 18 μm and that of senescent MSCs to be above 25 μm (Figure 5b). We then compared the enrichment efficiency by separating senescent MSCs from basal MSCs and captured them at outlet (iv) using 4‐μm and 13‐μm 3D filter arrays (Figure 5c). A control experiment was carried out by directly flowing the MSCs through the cell trap array to capture cells without a 3D filter. A significantly higher percentage (~65%) of SA‐β‐gal‐positive MSCs were found at the outlet (iv) for 13‐μm 3D filter array than the others. The result was consistent with those shown in Figure 3d, where more than 80% of the basal MSCs were able to pass through the 13‐μm 3D filter and recovered from outlet (iii). Therefore, we chose the senescence chip with a 13‐μm 3D filter array for selective removal of senescent MSCs from basal MSCs and blood components. At a flow rate of 3 ml/hr on this single‐unit small‐size chip, we achieved over 70% removal of prestained and SA‐β‐gal‐positive MSCs for both hydrogen peroxide and *X*‐ray‐induced senescent MSCs (Figure 5d).

Our results suggested that it was feasible to achieve preferential removal of senescent MSCs from undiluted whole blood without significant loss of basal MSCs using our senescence chip. However, the flow rate of 3 ml/hr is not high enough to process the large volume of human whole blood. To demonstrate the potential of our chips to achieve a higher throughput, we first scaled up the chip dimension by 10‐fold as shown in Figure 6a‐c. For example, the channel width was increased from ~10^3^ μm to ~10^4^ μm. To prevent the deformation and collapse of the wide PDMS channel under high flow pressure, we fabricated posts uniformly distributed inside the channel. Second, we stacked five devices along the vertical direction for parallel processing to further improve the throughput. The multiplexed chips shared the same inlets and outlets to keep the operation simple. Given the dominant hydrodynamic resistance by the filter array over inlets and outlets, uniform flow rates were applied on each of five chips. With this multiplexed system, the throughput was increased by two orders of magnitude from 3 ml/hr to 300 ml/hr. To characterize the performance of the ultrahigh‐throughput senescence chip, 4 × 10^4^ prestained MSCs containing both senescent and nonsenescent cells were spiked into 30 ml undiluted human whole blood for separation. As shown in Figure 6d and 6e, after separation, more than 70% of the senescent MSCs were removed while less than 15% of the basal MSCs were removed, demonstrating the similar performance from the high‐throughput multi‐unit chip compared to the single‐unit small‐size senescence chip.

Our study opens up great potential of our high‐throughput senescence chip in biological and clinical applications. Mesenchymal stem cells generally reside in the bone marrow, but small numbers can always be detected and isolated from peripheral blood. Certain cytokines increase MSC mobilization from the bone marrow to peripheral blood. For example, VEGF promotes mobilization of MSCs and is also a component of the senescence‐associated secretory phenotype (SASP) (Cashen, Link, Devine & DiPersio, 2004; Coppe et al., 2008; Pitchford, Furze, Jones, Wengner & Rankin, 2009; Rodier et al., 2009). Mesenchymal stem cell mobilization also increases under pathological conditions and hypoxia (Kuznetsov et al., 2001; Rochefort et al., 2006). By preferentially removing senescent MSCs directly from whole blood, we could minimize the negative health effects of their SASP, known to promote aging and tumorigenesis (Coppe et al., 2008; Rodier et al., 2009). Therefore, it is highly promising that with further development of our parallel multi‐unit senescence chip for an even higher throughput with robustness and biocompatibility, “senescence dialysis” could be developed to rapidly remove senescent cells in whole blood for anti‐aging therapy, similar to what “kidney dialysis” does for treatment of kidney failure by removing waste chemical from the blood (Rocco, 2007). Furthermore, higher rate (efficiency) of removal of senescent cells could be achieved by multiple iterations of full body blood circulation, as in the case of kidney dialysis.

Our platform may find wide applications where changes in cell sizes (either increase or decrease) can be utilized for the first step in cell enrichment, isolation, or removal. Although we focused on IR‐ or H_2_O_2_‐induced senescence of MSC in this study, other types of senescence including oncogene‐induced senescence (OIS) and therapy‐induced senescence (TIS), as well as senescence of other cell types (e.g., primary epithelial cells, senescent cancer cells), can be readily studied by our platform. For these studies, we will combine characteristic cellular markers specific for each cell type with senescence markers including SA‐β‐gal and SASP, to improve the specificity and reduce the false positives and false negatives resulted from the cell size difference alone. Furthermore, by optimizing the dimension of our 3D filter arrays and implementing multiscale size separation, we can achieve even higher sensitivity and efficiency for separation of cells with smaller size differences, for example, MSCs with gradually increased cell sizes at different stages of senescence, during the senescence progression.

## 3 CONCLUSIONS

In summary, we have developed senescence chips that incorporate novel 3D filter arrays for isolation, analysis, and removal of senescent cells from biofluids. After systematically characterizing the performance of our senescence chips with beads and cells, this device was applied to separate spiked human MSCs from whole blood for on‐chip cell analysis. We further validated its separation performance by isolating senescent cells from mouse bone marrow samples. Dose‐ and time‐dependent increases of the percentage of SA‐β‐gal‐positive MSCs were confirmed in our studies. To achieve rapid removal of senescent cells from human whole blood, we demonstrated a multi‐unit, large‐dimension senescence chip for parallel cell isolation, which could achieve the ultrahigh throughput at 300 ml/hr with more than 70% removal rate. This throughput could be further improved by incorporating more parallel units of 3D filter arrays, for potential biological and clinical applications, such as anti‐aging therapy by removing circulating senescent cells from human whole blood through “senescence dialysis.” We could implement multiscale filter arrays to reduce the loss of background white blood cells such as monocytes and macrophage in the same size ranges to further improve the sensitivity and specificity of our approach.

Compared to the existing microfluidic technologies, our platform has distinct advantages. It works directly with undiluted whole blood with no significant clogging due to the novel 3D filter design. Ultrahigh‐throughput size‐based cell separation in a continuous flow is achieved and this facilitates cell collection and downstream processing. This may enable subsequent omics‐level single‐cell analysis of the isolated senescent cells from small volumes of biological samples (Wang & Bodovitz, 2010). Compared to the conventional sieving approaches, cell damages could be minimized because target cells do not squeeze through the filtration pores but instead roll down the filter arrays during separation. Therefore, the isolated senescent cells could potentially be utilized for drug screening and assay development for anti‐aging therapies.

## 4 EXPERIMENTAL PROCEDURES

### 4.1 Device design and fabrication

The polydimethylsiloxane (PDMS) microfluidic chip was fabricated with soft lithography. The mask was designed with the AutoCAD software (Autodesk Inc., San Rafael, CA) and produced by Photo Sciences, Inc. (Torrance, CA). The silicon master as a PDMS mold was produced by standard photolithography and deep reactive ion etching (DRIE) techniques. For the parallel‐processing chip, five layers of identical PDMS channels were stacked up with the inlets and outlets aligned along the vertical direction.

### 4.2 Experimental setup

An epifluorescence microscope (IX83, Olympus, Japan) connected with a CCD camera (QIClick, QImaging, Canada) was used to observe and record the cell separation process inside the microfluidic channel. Two infusion syringe pumps (NE‐1600, New Era Pump Systems, USA; and KDS 100, KD Scientific, USA) were used to control flow rates. Due to the sedimentation of cells, the syringe containing blood sample was vertically positioned to ensure that most of the cells flowed into the microtubing. For the portable detection of MSCs on the chip, an iPhone 6 smartphone connected with a 60‐100x mobile phone microscope lens (Neewer, China) was used to take and visualize the images.

### 4.3 Sample preparation

Fresh human whole blood from healthy donors collected within 24 hr was purchased from AllCells Inc. (Alameda, CA). Human MSCs were purchased from Lonza (Lonza, Swiss). For hydrogen peroxide (H_2_O_2_) treatment, 30% H_2_O_2_ solution (Sigma, USA) were diluted with MSCs basal medium into desired concentrations. For *X*‐ray treatment, MSCs were placed on a rotating table and exposed to 1 Gy, 4 Gy, or sham (0 Gy), using a X‐RAD320 320 kVp X‐ray machine (Precision X‐ray Inc., North Branford, CT), operated at 300 kV, 10 mA (dose rate of 1.3 Gy/min). For mouse bone marrow samples, 10‐week‐old, male wild‐type mice (stain C57BL/6) were exposed to the total body X‐ray irradiation at 0 Gy (sham), 1 Gy, 4 Gy, and 6.5 Gy, with four mice at each dose, respectively. Mouse bone marrow samples were collected 10 days after TBI and diluted with 1 × PBS buffer to a total volume of ~1.5 ml per mouse. A Senescence Detection Kit (BioVision, CA) was used to stain senescent cells into blue color. PE‐CF594 mouse anti‐human CD‐45 antibodies (BD Horizon, USA) were used to label WBCs for fluorescence imaging.

### 4.4 Device operation

(i) Senescence chip for analysis of senescent cells in biofluids. The senescence chip with a 4‐μm 3D filter array and a cell trapping array was used to isolate MSCs from whole blood, capture MSCs on chip, and conduct single‐cell analysis in situ after capture. (ii) Senescence chip for removal of senescent cells from whole blood. A senescence chip with a 13‐μm 3D filter array was used to remove senescent MSCs from blood. A total of ~10,000 stained MSCs were spiked into 3 ml undiluted human whole blood and run through the device at a flow rate of 3 ml/hr. For the high‐throughput separation device, the flow rate was increased to 300 ml/hr. The removed senescent MSCs were collected in a tube from the outlet to measure the cell numbers and the percentage of senescent MSCs. All experiments were repeated at least three times.

### 4.5 Data analysis

(i) Quantification of senescent cells on cell culture plates. The MSCs were fixed and stained with Senescence Detection Kit and Hoechst 33342 (Thermo Fisher Scientific, USA). For each sample, five regions were randomly picked and recorded as a color image (RGB mode) and a fluorescent image (350/461, DAPI) using CCD camera on microscope with a 10x objective. The number of total MSCs and senescent MSCs was counted from the fluorescent images (DAPI) and color images (blue stain), respectively. (ii) Quantification of senescent cells on senescence chips. After staining of the MSCs on chip overnight, color images of MSCs were recorded with a CCD camera in RGB mode. The images were imported into ImageJ software to isolate their red channels, which were used to identify senescent MSCs. The grayscale of the dark region for each cell was measured with ImageJ, which define the senescent MSCs with a value smaller than 40.

More details for Materials and Methods are described in the Appendix S1.

## CONFLICT OF INTEREST

Y. C., P. M., and D. W. are employees of Newomics Inc., which might commercialize some of the technologies described in this work with pending patent applications.

## AUTHOR CONTRIBUTIONS

Y.C., P.M., and A.S. designed and performed experiments and wrote the manuscript. D.W. conceived and directed the experiments and wrote the manuscript.
