# Supplementary material for: Senescence chips for ultrahigh‐throughput isolation and removal of senescent cells
Source: Aging Cell. 2018 Jan 16;17(2):e12722. doi: 10.1111/acel.12722 (PMC5847876; doi:10.1111/acel.12722)
Supplement: Supplementary file 1 [file ACEL-17-e12722-s001.pdf]

Senescence Chips for Ultrahigh-throughput Isolation and Removal of Senescent Cells

Yuchao Chen,[a] Pan Mao,[a] Antoine M. Snijders,[b] and Daojing Wang*[a]

[a.] Newomics Inc., 5980 Horton Street, Suite 525, Emeryville, California 94608, United States

[b.] Lawrence Berkeley National Laboratory, Berkeley, California 94720, United States

AUTHOR INFORMATION

Corresponding Author

*Email: wang@newomics.com

MATERIALS AND METHODS

Device Design and Fabrication. The PDMS (polydimethylsiloxane) microfluidic chip was fabricated with soft lithography. The mask was designed with the AutoCAD software (Autodesk Inc., San Rafael, CA) and produced by Photo Sciences, Inc. (Torrance, CA). A simulation of the flow velocity inside the channel was conducted with COMSOL Multiphysics modeling software (Palo Alto, CA). The silicon master as a PDMS mold was produced by standard photolithography and deep reactive ion etching (DRIE) techniques. To fabricate the PDMS mold, 2 μm g-line photoresist (FujiFilm, USA) was coated on a 6-inch silicon wafer by spin coating. Followed with UV exposure to transfer the pattern from a mask to the photoresist layer, the silicon wafer was developed to generate a pattern on photoresist. After hard-bake, the wafer was etched by DRIE to produce channels with the desired depth. For the senescence chip, the channel depth was controlled between 30 and 35 μm. Finally, a Teflon layer was deposited on all surfaces of the silicon wafer to ensure a smooth PDMS peeling-off process. The senescence-chip was prepared by bonding the PDMS-replica channel onto a glass slide after treated with plasma (PDC-001, Harrick Plasma, USA). For parallel-processing chip, five layers of identical PDMS channels were stacked up with the inlets and outlets aligned along the vertical direction. Before use, the device was incubated at 65 °C overnight to prevent the fluid leakage and confirmed by the flow-through of 1x PBS buffer. Microtubing was connected to the PDMS channels for fluid delivery. The devices were disposed after each run of biological samples.

Experimental Setup. An epifluorescence microscope (IX83, Olympus, Japan) connected with a CCD camera (QIClick, QImaging, Canada) was used to observe and record the cell separation

process inside the microfluidic channel. The blood sample and 1x PBS buffer with 0.05% BSA were stored in syringes (BD Biosciences, USA). A 0.45 μm syringe filter (Acrodisc, Pall Life Sciences, USA) was connected to the syringe storing the PBS buffer to prevent contaminant from flowing into the microchannel and clogging the pillar array. Two infusion syringe pumps (NE-1600, New Era Pump Systems, USA; and KDS 100, KD Scientific, USA) were used to control the flow rates. When the senescence chip was tested with cells spiked in the undiluted whole blood, the buffer flow rate was usually 3 times of that of the blood sample. This ratio was decreased to 2 when the chip was tested with low concentration of cells or beads solution alone. Due to the sedimentation of cells, the syringe containing blood sample was vertically positioned to ensure that most of the cells flowed into the microtubing. When the senescence chip contains only one outlet with capture arrays for cell trapping, a longer microtubing was connected to the other outlet to balance the hydrodynamic resistance through both outlets. Before separation, 1x PBS buffer with 0.05% BSA flowed through the microchannel and microtubing for ~15 min to remove any remaining air bubbles and reduce nonspecific bonding to the channels.

Sample Preparation. Fresh human whole blood from healthy donors collected within 24 h was purchased from AllCells Inc. (Alameda, CA). The blood samples were collected with K2EDTA blood collection tubes (BD Biosciences, USA). Polystyrene beads with varied sizes were purchased from Polysciences, Inc. (Warminster, PA) and Bangs Laboratories, Inc (Fishers, IN). Coulter Z2 cell counter (Beckman Coulter, USA), BioRad TC20 cell counter (BioRad, USA), and a hemocytometer (Hausser Scientific, USA) were used to measure the number and concentration of cells and polystyrene beads and for cross validations. Human mesenchymal stem cells (MSCs) were purchased from Lonza (Lonza, Swiss). The log number used in this study was 0000471980

(derived from a 20-year-old male). MSCs were maintained in humidified incubators at 37 °C with 5% CO2, and cultured with MSCs basal medium (Lonza) supplanted with 5% FBS. MSCs at passage 6 were cultured on 12-well plates with proper densities to avoid over confluency over a 6-day period. The initial number of cells for each condition is shown in Table 1 and Table 2. For hydrogen peroxide (H_2O_2) treatment, 30% H_2O_2 solution (Sigma, USA) were diluted with MSCs basal medium into desired concentrations. Media containing 100 μM and 200 μM H_2O_2 as well as the basal medium control were used to incubate MSCs at 37 °C for 2 h. After that, the MSCs were washed with 1x PBS solution 3 times and cultured in the fresh media for another 3 days before analysis. For X-ray treatment, MSCs were placed on a rotating table and exposed to 1 Gy, 4 Gy or sham (0 Gy), using a RAD320 320 kVp X-ray machine (Precision X-ray Inc., North Branford, CT), operated at 300 kV, 10 mA (dose rate of 1.3 Gy/min). Cells were cultured for another 3 days and 6 days, respectively, before analysis.

Table 1. Initial number of MSCs on a well for hydrogen peroxide treatment

	0 μM	100 μM	200 μM
3 Days	1×10^4 cells/well	1.5×10^4 cells/well	2×10^4 cells/well

Table 2. Initial number of MSCs on a well for X-ray irradiation

	0 Gy	1 Gy	4 Gy
3 Days	1×10^4 cells/well	2×10^4 cells/well	4×10^4 cells/well
6 Days	5×10^3 cells/well	1×10^4 cells/well	2×10^4 cells/well

For mouse bone marrow samples, 10 weeks old, male wild-type mice (stain C57BL/6) were exposed to the total body X-ray irradiation at 0 Gy (sham), 1 Gy, 4 Gy, and 6.5 Gy, with 4 mice at

each dose, respectively. The bone marrow samples were collected 10 days after the X-ray treatment and diluted with 1x PBS buffer to a total volume of ~1.5 mL per mouse.

For cell separation study, MSCs were dissociated with trypsin (Lonza, Swiss), fixed, and stored in 1x PBS buffer. Before being processed with a senescence chip, the whole blood sample spiked with MSCs were filtered with a 40 μm cell strainer (Falcon, Corning, USA) to remove contaminants and clotting. To identify senescent MSCs, a Senescence Detection Kit (BioVision, CA) was used to stain senescent cells into blue color. To stain suspended MSCs, cells were incubated with the staining solution on chip or inside a tube at 37 °C overnight before study.

For WBC study, 10 mL human whole blood was added to 200 mL 1x RBC lysis buffer (BioLegend, San Diego, CA) and incubated at room temperature for 15 min, followed by a centrifugation at 350 × g for 5 min to enrich WBCs. The isolated WBCs were resuspended with 10 mL 1x PBS buffer and used to characterize our senescence-chips. The concentration of the input WBCs and recovered WBCs was measured with a Bio-Rad cell counter. To differentiate WBCs from the RBCs background, the nucleus of WBC was stained with Hoechst 33342 (Thermo Fisher Scientific, USA) and observed under a fluorescence microscope (350/461, DAPI).

Device Operation

Senescence chip for analysis of senescent cells in biofluids. The senescence chip with a 4 μm 3D filter array and a cell trapping array was used to isolate MSCs from whole blood, capture MSCs on chip, and conduct single cell analysis in situ after capture. 2 mL of fresh undiluted human whole blood spiked with ~500 fixed senescent MSCs induced by either H_2O_2- or X-ray was injected into the senescence-chip at a flow rate of 3 mL/h. For mouse bone marrow samples, we aliquoted ~1 x 10^6 bone marrow mononuclear cells (BM-MNCs) from each sample. The aliquots were diluted

into 2 mL with 1x PBS before loading directly on our chips for cell separation. 1x PBS buffer with 0.05% BSA was injected from another inlet at a flow rate of 9 mL/h. After MSCs were captured on the cell trapping array, the flows of cell sample and buffer were stopped, and followed by a gentle injection of staining solution to fill the whole channel and tubing. The inlet tubing was kept during incubation to generate a balance pressure and prevent backflow of the trapped cells. During the separation and staining processes, care was taken to avoid air bubbles inside the channel. After incubation, color images of the captured MSCs were recorded with the microscope for analysis. All the experiments were repeated at least 3 times.

Senescence chip for removal of senescent cells from whole blood. A senescence chip with a 13 μm 3D filter array was used to remove senescent MSCs from blood. Before spiked into human whole blood, the fixed senescent MSCs induced by either H_2O_2 or X-ray were stained overnight with the Senescence Detection Kit in a centrifuge tube. The percentage of the senescent MSCs was manually counted under the microscope by dropping 10 μL of the stained MSC sample on a glass slides. Then ~10,000 stained MSCs were spiked into 3 mL undiluted human whole blood and run through the device at a flow rate of 3 mL/h. For the high-throughput separation device, the flow rate was increased to 300 mL/h. The removed senescent MSCs were collected in a tube from the outlet to measure the cell numbers and the percentage of senescent MSCs. The number of input and output senescent cells were calculated accordingly. The removal rate was then determined by the ratio of output senescent MSCs over to input senescent MSCs. All experiments were repeated at least 3 times.

Data Analysis

Quantification of senescent cells on cell culture plates. To quantify the senescent MSCs on cell culture plates, the MSCs were fixed and then stained with Senescence Detection Kit and Hoechst 33342 (Thermo Fisher Scientific, USA). For each sample, five regions were randomly picked and recorded as a color image (RGB mode) and a fluorescent image (350/461, DAPI) using CCD camera on microscope with a 10x objective. The number of total MSCs and senescent MSCs were manually counted from the fluorescent images (DAPI) and color images (blue stain), respectively. Therefore, the percentage of senescent MSCs in each sample was determined by the ratio of senescent MSC number to total MSC number.

Quantification of senescent cells on senescence chips. After staining the MSCs on chip overnight, the color images of MSCs were recorded with a CCD camera in RGB mode. Microscope lamp intensity was consistent at 5 V. The images were then imported into ImageJ software to isolate their red channels, which were used to identify senescent MSCs. The grayscale of the dark region for each cell was measured with ImageJ, which define the senescent MSCs with a value smaller than 40.

RESULTS AND DISCUSSION

Design and working mechanism of senescence chips. We first developed the senescence chip which monolithically integrate two rows of tilted 3D filter array for size-based cell separation with all necessary inlets and outlets for samples and buffers (Figure 1). Two types of senescence chips were designed for different purposes. For analysis of senescent cells in small volumes of whole blood or bone marrow, the senescence chip contains a 3D-filter array to isolate MSCs, followed with a cell trap array to capture MSCs after separation for enumeration and single cell analysis of

senescent cells (Figure 1a-i). For rapid removal of senescent cells from whole blood, the senescence chip does not contain cell traps but the chip outlet is connected directly to a tubing to remove senescent cells from whole blood (Figure 1a-ii). The other end of the tubing goes to a waste or a collection tube for further analysis if needed.

We performed modeling to optimize the design of our chips (Figure 1b). A 3D filter array contained PDMS micropillars inside a channel to achieve cell separation on the x-y plane as well as in the z direction. On the x-y plane, two key parameters were taken into consideration, which were the inclination angle (θ) of micropillars relative to the main fluidic flow, and the inter-pillar spacing (d) as shown in Figure 1b-i. The pillar shape was also optimized to minimize clogging and maximize cell separation. Two types of quadrangle pillars were designed as shown in the zoom-in of Figure 1a. When moving down on the filter arrays, the particle tends to be trapped by the sharp edge of the Type-A pillars. In contrast, the particle contacts a tilted surface on Type-B pillars, which is easier to move on. Therefore, the Type-B pillars show a better performance in particle separation. For the rigid particles with diameters smaller than the pillar spacing (d), they could directly pass through the filter. When a particle has a diameter larger than the pillar spacing, we divided the hydrodynamic drag force (F) into two portions, parallel to the filter (inclination plane, F_1) and perpendicular to the filter (F_2). To ensure the particle could roll down on the filter, the relation

$$F_1 \bullet L_1 > F_2 \bullet L_2 \qquad (1)$$

should be established, in which L_1 and L_2 are the arms of forces F_1 and F_2. In Eq. (1), F_1 and F_2 could be expressed as F•cosθ and F•sinθ, while L_1 and L_2 could be expressed as $(R2\text{-}1/4d2)^{1/2}$ and 1/2d, in which R is the radius of the particle. Therefore, Eq. (1) could be expressed as

$$d < 2R/(\tan 2\theta + 1)^{1/2} \qquad (2)$$

From Eq. 2, a smaller pillar spacing (d) and filter angle (θ) would help particles to roll down on the pillars as shown in Figure 1a. In our design, we set the angle of filter array (θ) at 5°. The pillar spacing (d) (also called “filter size” hereafter) is varied based on the size of cells to be isolated.

In the z direction, the PDMS pillars do not bond to the glass substrate because of their small top-surface area. Therefore, depending on the operational flow rate in the channel, an opening with varying size is created between the pillars and the glass substrate, which works like a shutter and allows smaller cells to pass through (Figure 1 b-ii and iii). For example, during the separation of MSCs from whole blood, RBCs and WBCs can easily pass through the filter from both the z-direction and x-y plane, while the MSCs with a larger size will not cross the filter but instead roll down. Our simulation shows that, compared to the 2D filter array (Figure S1, a and c), 3D filter array (Figure S1, b and d) can generate much more uniform flow velocity across the channel. Therefore, the design of our 3D filter array could better reduce the system backpressure, reduce clogging of the filter, and improve the throughput.

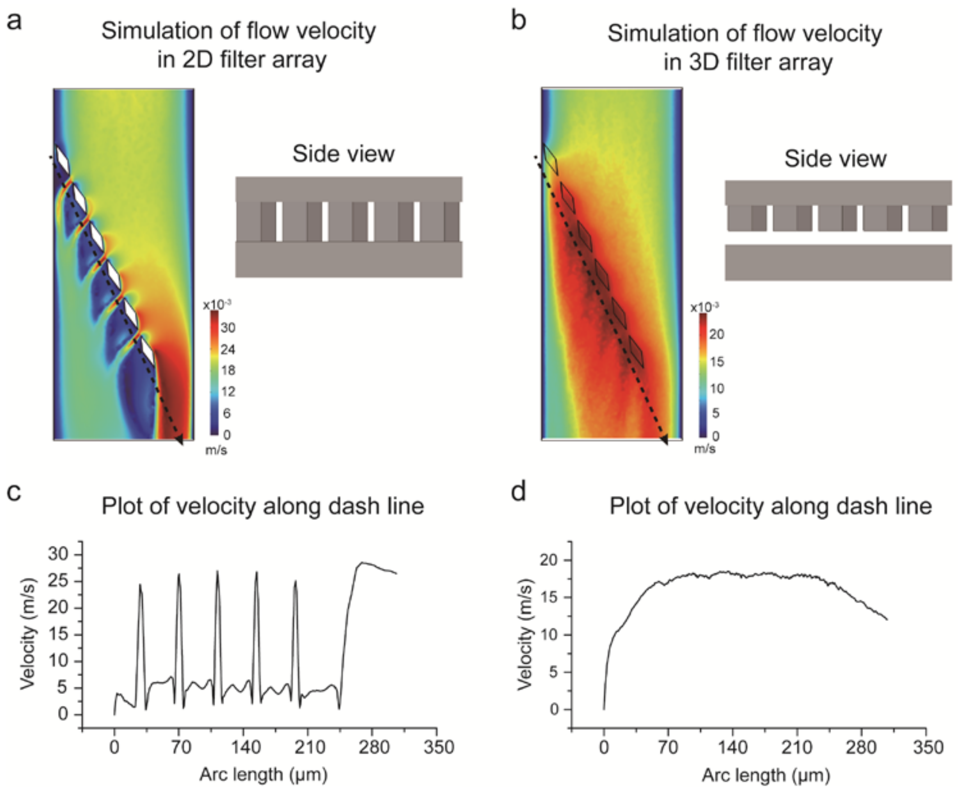

Figure S1. Flow simulation inside the microfluidic channel. (a) Simulation of flow velocity in a 2D filter array. (b) Simulation of flow velocity in a 3D filter array. (c) Plot of flow velocity along the dash line in (a). (d) Plot of flow velocity along the dash line in (b).
